# VarRanker: rapid prioritization of sequence variations associated with human disease

**DOI:** 10.1186/1471-2105-14-S13-S1

**Published:** 2013-10-01

**Authors:** Brendan D O'Fallon, Whitney Wooderchak-Donahue, Pinar Bayrak-Toydemir, David Crockett

**Affiliations:** 1 ARUP Institute for Clinical and Experimental Pathology, 500 Chipeta Way, Salt Lake City, UT, USA

## Abstract

**Background:**

Identification of the genetic alterations responsible for human disease is a central challenge facing medical genetics. While many algorithms have been developed to predict the degree of damage caused by a given sequence alteration, few tools are able to incorporate information about a given phenotype of interest.

**Methods:**

Here, we describe an algorithm and web-based application which take into account both the probability that a variant damages the function of a gene as well as the relevance of the gene to a given phenotype. Phenotypes are described by a list of scored terms supplied by the user. These terms are then used to search a variety of public databases including NCBI gene summaries, PubMed abstracts, and Gene Ontology terms, and protein-protein interactions in String-DB to determine a relevance score. The overall ranking is determined by the product of the functional damage score and the relevance score, such that highly ranked variants are likely to be damaging and in genes of interest.

**Results:**

We demonstrate the method on several test cases including samples with Hereditary Hemorrhagic Telangiectasia (HHT) and Diamond-Blackfan Anemia (DBA). We have also implemented a web-based application which allows public access to the VarRanker algorithm.

**Conclusions:**

Automated searching of public literature and online databases may substantially decrease the amount of time required to identify the mutations underlying human disease. However, several ad-hoc and subjective decisions must be made, and the results of such analyses are likely to depend on the researcher and the state of the literature and databases involved.

## Background

A fundamental challenge facing medical geneticists is the efficient ranking and filtering of genetic sequence variations by their likelihood of association with a particular phenotype. Newly developed high-throughput sequencing technologies quickly identify thousands to millions of sequence variants per sample, and while common filtering strategies may substantially reduce this number, often hundreds or thousands of potentially interesting variants must be manually investigated to determine their relevance to the phenotype under consideration. This process may be very time consuming even for a single sample, and manual examination is likely to be impractical for larger, multi-sample studies [1].

Many algorithms have so far been developed to classify genetic variants by their probability of causing disease (e.g. [2][3][4]-[5]). These classification strategies utilize multiple sources of information to determine the probability that a given variant is damaging, including sequence conservation, the biochemical properties of the amino acids involved, and the characteristics of known-disease causing mutations culled from the literature. While these tools are valuable for gene discovery studies, their usefulness is limited by several factors. First, all individuals, including healthy individuals, bear a large number of variants predicted to be disease-causing. Thus, while some fraction of variants may be filtered out because they are predicted to be benign, researchers may nonetheless be confronted with hundreds of damaging variants per sample. Second, the strategies do not incorporate phenotypic information regarding the condition in question and predict only the probability that a variant disrupts the function of a protein in question, and not whether the protein influences a particular phenotype.

Appropriate incorporation of phenotypic information into variant classification strategies is complicated by several factors. First, while numerous public databases exist that contain information about genes and their related phenotypes, the databases vary widely in the type, quality, and update frequency of the information. Efficiently retrieving and parsing the relevant information from these sources represents a nontrivial technical obstacle. Second, many options exist for gathering phenotypic information supplied by the user. Previously described strategies require a disease name or set of training genes to generate results (e.g.[6][7][8]). These methods use a variety of similarity measures, including sequence similarity, protein-interaction network features, and Gene Ontology terms to identify genes with similarities to the target set. Finally, converting the information supplied by the user and that obtained from the databases into a raw numerical score presents several challenges, in particular how heavily to weight various subscores and how to combine scores from disparate measures. Overcoming these challenges is likely to involve some ad-hoc decisions, and the algorithm's output will reflect the subjective nature of the input as well as the current state of the databases queried. Despite the subjective nature of such procedures many variants may be rapidly eliminated based on their lack of association with any term, gene, or process of interest.

Here, we propose a variant prioritization algorithm that explicitly includes both the probability that a given variant damages the gene's function as well as a broad measure of the relevance of the gene to a given phenotype. The product of these two orthogonal measures is then taken as a predictor of the overall likelihood that a given variant is responsible for some phenotype under consideration. Phenotypic information is obtained from four sources: NCBI gene summaries, PubMed abstracts, Gene Ontology (G.O.) terms, and protein-protein interactions. By using two largely independent measures of relevance we show that the list of variants requiring manual inspection may be substantially shortened, facilitating rapid identification of the sequence variations underlying a given phenotype.

## Methods

The algorithm combines two orthogonal measures to rank variants. The first measure combines the scores of six different mutation effect prediction algorithms into a single value that quantifies the probability that the variant damages the function of the gene in which it resides. We term this score the 'functional damage score'. The second measure examines each gene and quantifies its relevance to a particular phenotype, where the phenotype is described by the user using several techniques. We refer to this score as the 'relevance' score. Details of both methods are described below. Each method produces a single value quantifying the contribution of the method. The product of the two values determines the overall rank of a particular variant.

### Quantification of functional damage

We assume that for a genetic variant to cause a disease phenotype it must disrupt protein function in some way. To assess the degree to which a variant disrupts protein function we combine six previously described variant classification methods into a single score. The six scores are SIFT [2], Polyphen-2 [3], MutationTaster [4], GERP++ [9], PhyloP [10], LRT [11], and SiPhy [12], and we use a table of precomputed scores for all amino-acid changing positions in the human genome described in dbNSFP2.0 [13]. The scores encompass a variety of variant classification strategies, including evolutionary conservation, sequence properties such as CpG content, and amino acid properties such as size and hydrophobicity. Intronic, intergenic, and silent changes are ignored.

To combine the six measures into a single score, we sought a linear combination of scores that produces the maximal difference between variants known to be disease-causing and those unlikely to cause disease. Previous studies have shown that combining multiple prediction algorithms can lead to greater overall sensitivity and specificity [14][15]. To obtain a data set of known disease-causing mutations we consulted those described in the Human Gene Mutation Database [16], extracted only those mutations categorized as disease causing, and disregarded all functional polymorphisms and variants without support from functional studies (disease-associated polymorphisms). The total number of disease-causing mutations was 14,264. For the non-disease associated mutations we extracted all SNPs at greater than 5% frequency in the 1000 Genomes Project, Nov. 2010 release, totalling 7,325 SNPs. For a given set of coefficients w, we are then able to compute for any position and mutational change a single score that is a linear combination of the six prediction algorithms using the following equation:

(1) $$S_{i}=\overset{6}{\underset{j=1}{\sum }}w_{j}x_{j}$$

where *S_i _*is the "combined score" of variant *i*, *w_j _*is the weight given to prediction algorithm *j*, and *x_j _*is the score of the prediction algorithm *j *for variant *i*. We sought to identify the set of weights w* that maximized the difference between the disease-causing and the non-disease associated mutations. To accomplish this we constructed an ad-hoc function that quantified the amount of similarity in the distributions of scores for the two data sets, as follows:

(2) $$Z=\frac{\sigma_{a}+\sigma_{b}}{\bar{a}-\bar{b}+c}$$

**Figure 1:**
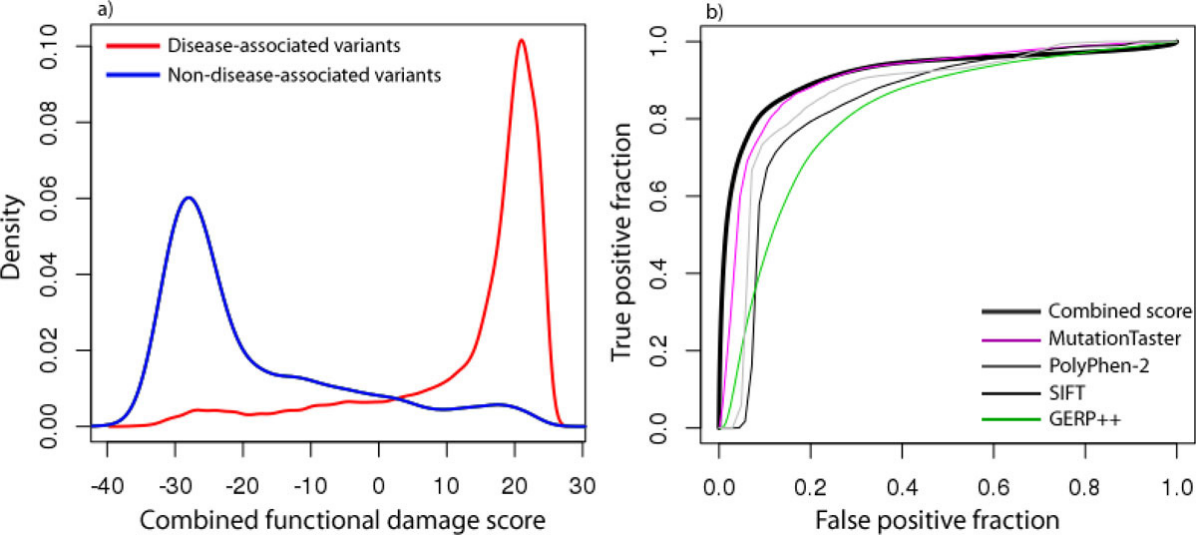
**Combined functional damage scores for disease and non-disease associated variants**. Figure 1) a) Distribution of functional damage scores for mutations previously associated with disease (red line) and those at > 20% frequency in 1000 Genomes (blue line). b) ROC curve for functional damage score compared to selected other predictors.

Where σ_a _and  ā are the standard deviation and mean of the scores for all disease-causing mutations, respectively, with similar nomenclature for non-disease associated mutations, and *c *is a small constant (0.001) that prevents the denominator from attaining very small values when the mean of *a *and *b *are nearly equal. We then employed a numerical function minimization technique to determine the set of weights that resulted in the smallest possible *Z*, which is associated with the greatest difference in means and smallest standard deviations among both sets of variants. The resulting distribution of combined scores clearly separates the disease-causing mutations from those that were assumed to be non-disease causing (Figure 1a). Receiver operating characteristic (ROC) curves for the combined score demonstrated greater sensitivity and specificity than for any single predictor alone (Figure 1b) and yield satisfactory false discovery rates for a given degree specificity (Table 1).

**Table 1:** Sensitivity and Specificity of Functional Damage

Threshold	False positives	True positives
0	21.2%	89.5%
5	16.9%	86.3%
10	13.5%	85.1%
15	10.7%	82.8%
20	8.5%	81.1%

**Table 2:** Ranked Variants for HHT sample

Variant	Functional Damage score	Relevance Score	Overall score
RASA1 (p.87_88del)	25	42.9	1072.5
AGTR1 (p.A244S)	17.5	10.5	184.3
ENPEP (p.R925G)	15.8	9.5	150.0
MYOD1 (p.A162V)	15.7	8	126.2
DBH (p.A362V)	17.1	6.8	116.9

Finally, we note that the precomputed scores available in dbNSFP2.0 are not available for indels and do not take into account effects of variants on mRNA splicing. We arbitrarily assigned a score of 25 to all frameshifting indels and 15 to all variants in splice sites, predicting that both classes of mutations are likely to be more functionally damaging than most SNPs. Nonframeshifting indels are given a score equal to their length up to a maximum of 15.0.

### Quantification of relevance of gene to phenotype

The second component of the algorithm quantifies the probability that a given gene is related to a phenotype of interest by examining and combining information from four publicly available databases. Specifically, we examine NCBI gene summaries, Gene Ontology (G.O.) terms, PubMed abstracts, and gene interaction networks for information. The first three databases are obtained directly from the NCBI, while gene interaction networks are obtained from the STRING database (version 9.0)[15]. Phenotypic information is provided by the user in three forms, scored key terms, scored G.O. terms, and genes of interest.

The scored key terms are a list of arbitrary terms associated with a numerical score reflecting their relevance to the phenotype, and are used to examine both gene summaries and pubmed abstracts. For each NCBI gene summary examined (typically all genes in which a variant exists are examined), the entire text of the summary is scanned to determine if query term exists. If so, the score associated with the term is added to the 'summary score' for that gene. Multiple hits of the same term do not add increased scores. The PubMed abstracts associated with a given gene are obtained from the 'gene2pubmed' database maintained by the NCBI. For each gene examined all abstracts as well as article titles are obtained from the NCBI. All NCBI files were obtained from ftp://ftp.ncbi.nlm.nih.gov/gene/DATA. In a manner similar to the gene summary procedure the entire text of the abstract as well as the title of the article is examined for the presence of the term. If the term exists in the abstract or summary the score associated with that term is tallied and the total score for each abstract is computed. The top-scoring abstract for each gene is then retained as the 'abstract score' for the gene.

The G.O. term search procedure mirrors the gene summary procedure closely. For each gene considered G.O. terms are obtained from NCBI databases and are scanned for the presence of the user-supplied G.O.terms. If a match is found, the score associated with the term is tallied and added to the 'G.O. Score' for the gene.

Gene interaction information is obtained from the STRING database [17]. Users supply a list of genes known or strongly suspected to play a role in the phenotype of interest, which we refer to as 'seed genes'. These genes are used as the query genes in a STRING database query, and the 'neighborhood' of surrounding genes is obtained by expanding the network size around the seed genes until the network contains 500 genes. Network expansion is accomplished by altering the 'additional network nodes' parameter within STRING-DB. The raw output of the STRING procedure is a list of interactions and their associated confidence scores. This list is converted to a graph, where each gene is represented by a node and each interaction is a weighted edge. For every gene in the graph we compute the shortest path to each of the user-supplied query genes using Dijkstra's algorithm. Because weights represent stronger, not weaker, interactions, the reciprocal of each weight is used to determine path length. The shortest path leading to any key gene is then retained as the final 'interaction score' for each gene.

The above procedure computes for each gene under consideration four independent scores, one each for gene summaries, PubMed abstracts, G.O. terms, and gene interactions. The sum of these scores then represents the total relevance of the gene to the phenotype supplied by the user. The score is necessarily ad-hoc, and may vary greatly by the subjective description and scores given for each term. In addition, gene relevance scores will change over time due to updates of gene summaries, the appearance of new abstracts in PubMed, or new interactions recorded in the STRING database.

## Results

To assess the ability of our method to accurately identify disease-causing variants we analyzed three sets of data in which causative variants had been previously identified. Because our method relies heavily on literature searches and the knowledge available in public databases, we chose data sets with differing degrees of prior knowledge, as measured by the number of articles retrieved in PubMed (as of August, 2012) when searching for the name of the condition. The least well studied condition we examine is Miller Syndrome, for which 33 citations exist in PubMed. The most well studied data set is Hereditary Hemorrhagic Telangiectasia (HHT, 2534 articles), and an intermediate case is Diamond-Blackfan Anemia (DBA, 303 articles).

### A. Analysis of Miller syndrome variants

Miller syndrome (also known as postaxial acrofacial dysostosis, OMIM #263750), a rare autosomal genetic disorder characterized by micrognathia, cleft lip or palate, hypoplasia of the posterior portions of the limbs, and colobma of the eyelids, was one of the first disorders in which a causative gene was identified by exome sequencing [18]. Four variants were initially uncovered by exome sequencing, and several others identified by followup Sanger sequencing in additional individuals. The causative gene was determined to be *DHODH *on the basis of variant rarity, damaged function predicted by the PolyPhen tool, and consistent inheritance pattern across kindreds.

To determine the ability of our method to recapitulate these known causative variants, we examine exome variant sets into which the Miller syndrome variants were added. The Miller syndrome variants represent a difficult case for the tool described here since relatively little is known about the pathogenesis of the disorder (excluding [18]). Thus little phenotypic information exists to inform a guided analysis. The absence of any previously identified genes or pathways affected in Miller syndrome precludes the use of both the G.O. term and genetic interaction subscores. Nonetheless, to demonstrate the flexibility of the algorithm in the face of incomplete data we utilize only the gene summary and PubMed abstract subscores in our analysis. A complete listing of search terms and scores is in supplementary table I.

To determine the ranking of the Miller syndrome variants we spiked the variants into a control background of exome data from the widely studied sample NA12878 (CEPH, Utah resident). The full set of input terms is listed in supplementary material. After conducting our ranking procedure, the seven Miller syndrome variants occupied positions 9-14 and 17-18 out of a total of 26,670 exonic variants, 11,969 of which were nonsynonymous, indels, or splice-site variants. The composite gene-damaging score was very high for all *DHODH *variants, ranging from 13.4 - 20.5, with scores above 20 for five of the eight variants examined. At a cutoff of 20, the gene damage score is greater than 98.9% of all variants in the sample for which a score could be computed. The gene relevance score was more moderate, however, with many non *DHODH *genes associated with cleft lip and cleft palate.

### B. Analysis of Hereditary Hemorrhagic Telangiectasia sample

To demonstrate our algorithm on a case where more complete phenotypic information is available, we examine a data set obtained from a patient with Heredity Hemorrhagic Telangiectasia (HHT, OMIM #187300), an autosomal dominant disorder with clinical presentation including spontaneous recurrent epistaxis (nosebleeds), telangiectasias, and arteriovenous malformations (AVMs) in the brain, liver, spinal column, and lungs [19]. Mutations in the ENG, ACVRL1, and SMAD4 genes are responsible for the disorder in some 80% of cases, but the genetic causes are unknown in the remaining fraction. While understanding of the molecular and cellular processes underlying AVM formation in HHT patients is incomplete, a substantial literature exists regarding the mechanisms, pathways, and genes involved in angiogenesis. Thus, ample information exists to create gene summary, PubMed, and G.O. term lists as well as a gene interaction network targeting the relevant genetic neighborhood.

Recently, members of our lab identified a frameshifting 2bp deletion in *RASA1 *(p.87_88del) in an individual presenting with HHT-like symptoms. Because RASA1 mutations have previously been associated with capillary and arteriovenous malformations similar to the HHT phenotype ([20][21]), and because no variants were identified in the ENG, ACVRL1, or SMAD4 genes, the RASA1 variant was assumed to be the causative mutation for this sample. We re-analyzed this sample using our algorithm to determine if the causative gene would be correctly identified. Variants with frequency 1% or greater among the 1000 Genomes samples were excluded. All search terms and seed genes used as input are listed in supplementary tables II and III.

The top five ranking variants are shown in table II. The RASA1 variant is the overall top scoring candidate, with a combined score roughly five times greater than the next candidate. Frameshifting deletions are regarded as very likely to be damaging in terms of genetic function by our algorithm, and both gene summaries and PubMed abstracts strongly suggest that RASA1 mutations may have phenotypic effects similar to those found in HHT.

The very high score computed for the RASA1 variant relative to other variants in the sample demonstrates that, when ample prior knowledge is available, our algorithm can successfully identify disease causing variants.

### C. Analysis of Diamond-Blackfan anemia sample

As an additional example we examined a splice-site variant in the GATA1 gene was that recently shown to cause Diamond-Blackfan anemia (DBA, OMIM #105650) [22]. As mentioned above, DBA an intermediate amount of prior knowledge exists for DBA, with roughly 300 citations associated with the condition found in PubMed. DBA is an erythroid aplasia resulting from inadequate proliferation of erythroid progenitor cells with a population incidence near 5 in one million live births. Previous studies have implicated mutations in ribosomal protein RPS19 as causing DBA in roughly 25% of cases [23][24], and eight other ribosomal proteins have been implicated in additional studies [25].

We were unable to access the full variant lists for the affected individuals, so we inserted the causative mutation into a variant listing from the well-studied individual NA12878. Variants with frequency 1% or greater among the 1000 Genomes samples were excluded, and all search terms and seed genes used in the analysis are given in the supplementary tables IV and V. The top five overall ranking variants are given in the Table 3.

**Table 3:** Ranked Variants for DBA sample

Variant	Functional Damage score	Relevance Score	Overall score
GATA1 (p.V74L)	15	99.28	1489.30
ICAM1 (p.G241R)	24.24	45.58	1105.33
FCGR2A (p.Q62X)	20	42.02	840.43
ERCC4 (p.R415Q)	17.87	46.83	836.96
GYPA (p.E13A)	15	54.09	811.46

Our algorithm successfully identified the GATA1 variant as the top-ranked variant, although the separation of the causative variant from other variants is smaller than for the RASA1 case. All splicing variants are arbitrarily assigned a functional damage score of 15.0, thus the true variant is predicted have to fairly severe functional consequences. Despite this large score, some 830 other variants had a score as high or higher. However, the GATA1 gene also has a very high overall relevance score, and the product of the two relatively high scores was greater than all other variants.

## Conclusions

Our method facilitates filtering and prioritization of genetic sequence variants by their degree of association with a phenotype. By utilizing two orthogonal measures of variant importance, the algorithm can significantly reduce the number of variants requiring manual consideration and thereby allow researchers to more rapidly identify the sequence variations underlying a given disease. In addition, the algorithm's multi- database, search-term based approach allows for substantial flexibility in terms of the phenotype associations it can detect, and need not be employed strictly for the study of human disease.

While we have presented several successful cases here, the method's ability to accurately identify causative variants is influenced by a number of factors. Most importantly, our method is sensitive to the state of the literature and public databases queried, and conditions about which relatively little is known (such as Miller Syndrome) are less likely yield successful results with our method. In addition, the user's subjective choice of input terms and their associated scores may lead to results that differ form researcher to researcher. Finally, our method is designed primarily for Mendelian disorders, where one or a few variants of large effect are responsible for producing the condition. Phenotypes that are influenced by a larger number of interacting, compensatory, or weak-effect mutations will be difficult to analyze with the framework presented here.

**Figure 2:**
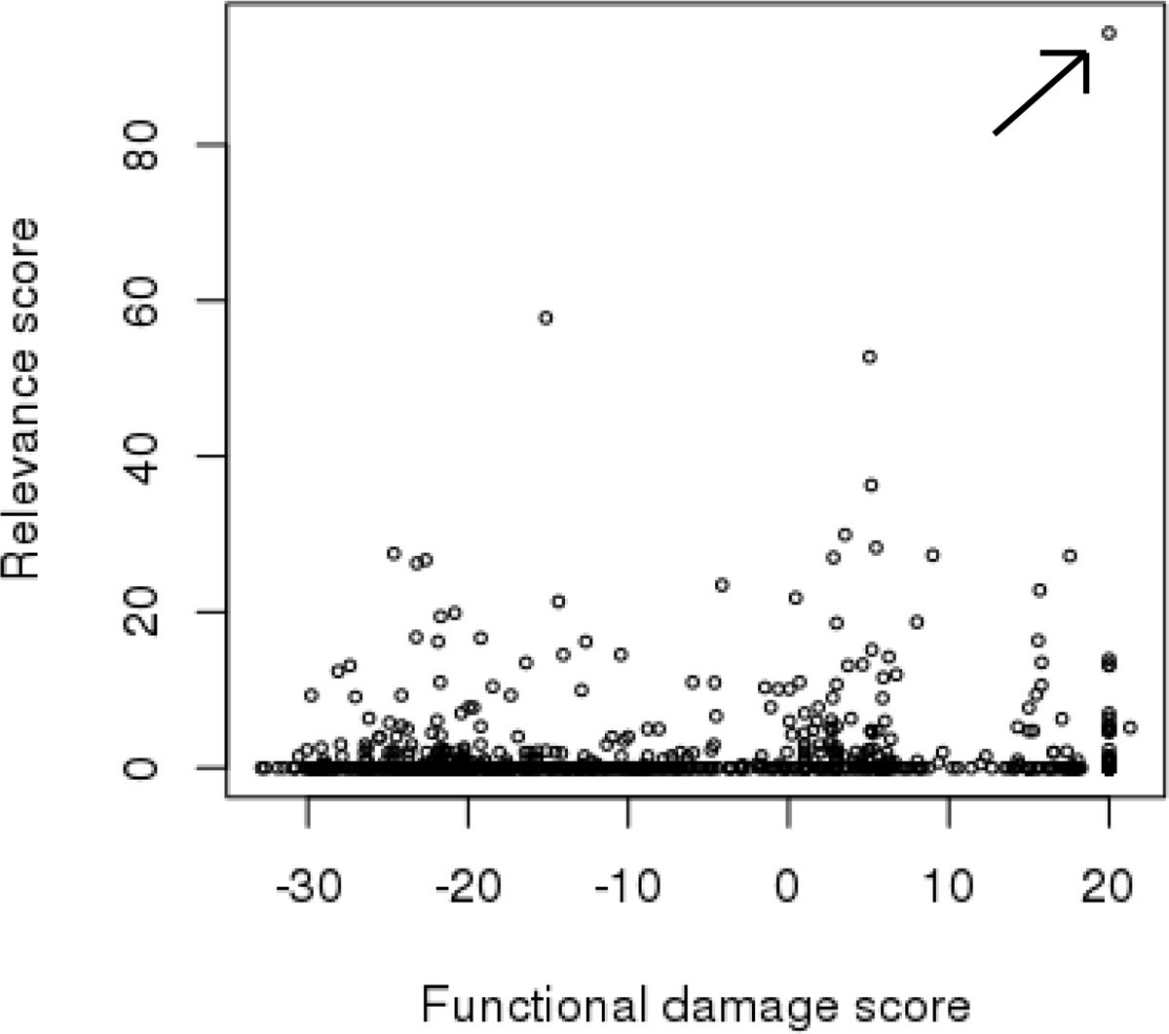
**Distribution of variant scores in HHT example case**. Figure 2: Distribution of variants by functional damage score and gene relevance score for HHT sample. Arrow indicates causative frameshift in RASA1 gene.

**Figure 3:**
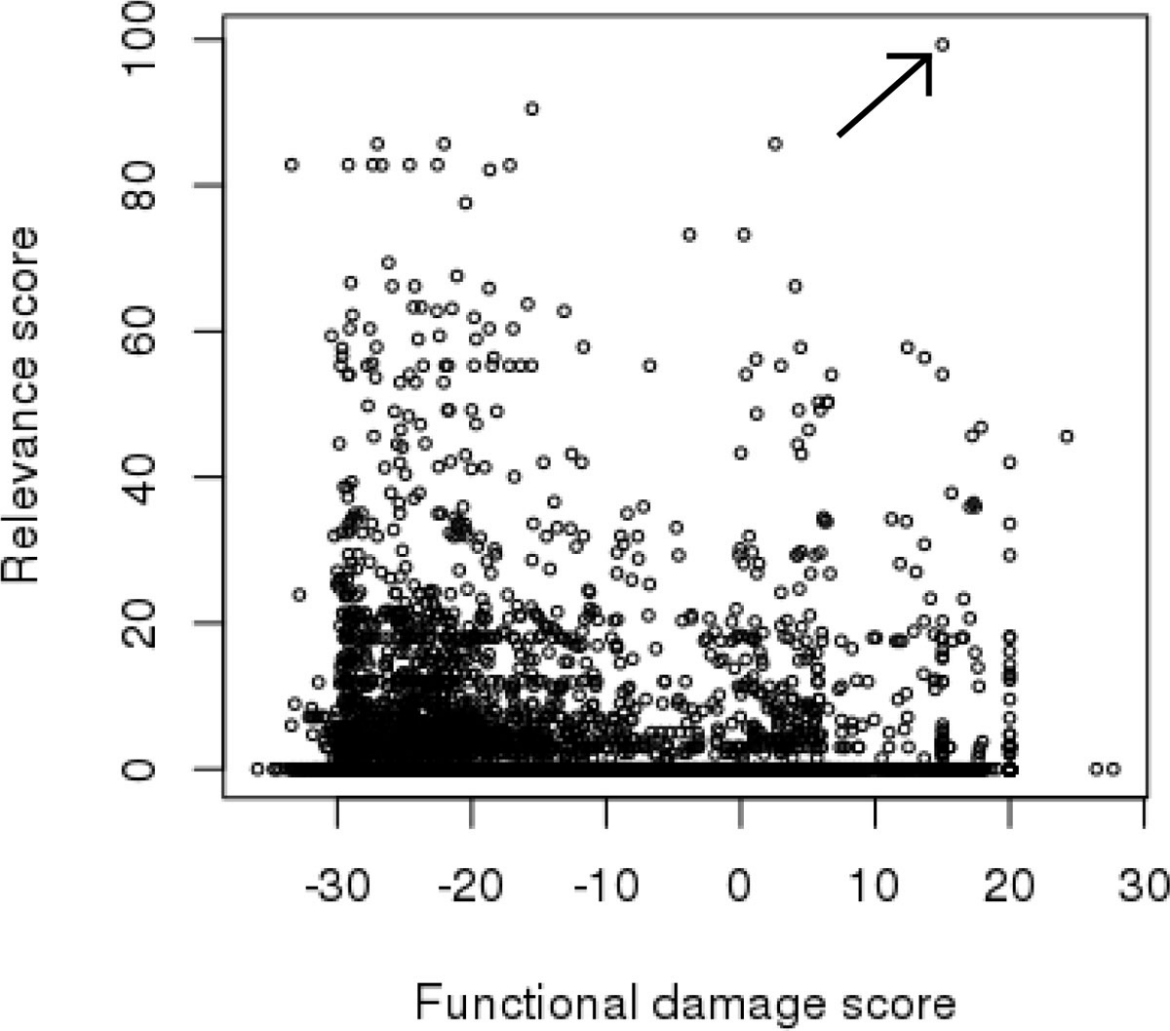
**Distribution of variant scores for DBA example case**. Figure 3: Distribution of variant scores for Diamond-Blackfan Anemia analysis. Arrow indicates causative mutation in GATA1.

## Competing interests

The authors declare that they have no competing interests.

## Authors' contributions

BDO designed and implemented the algorithm, wrote the paper, and conducted the analysis. WWD and PBT and contributed data regarding the HHT example case.
